# An easy method to generate recombinant pseudorabies virus expressing the capsid protein of Porcine circovirus type 2d

**DOI:** 10.3389/fmicb.2023.1206021

**Published:** 2023-05-31

**Authors:** Jingqiang Ren, Rachel Madera, Chase Cunningham, Jishu Shi, Lihua Wang

**Affiliations:** ^1^Department of Anatomy and Physiology, College of Veterinary Medicine, Kansas State University, Manhattan, KS, United States; ^2^Institute of Virology, Wenzhou University, Chashan University Town, Wenzhou, China; ^3^Key Laboratory of Special Animal Epidemic Disease, Ministry of Agriculture, Institute of Special Economic Animal and Plant Sciences, Chinese Academy of Agricultural Sciences, Changchun, China

**Keywords:** pseudorabies virus, homologous recombination, linearization, efficient, PCV2d

## Abstract

**Introduction:**

Homologous recombination is an effective way to generate recombinant viruses for vaccine research such as pseudorabies virus (PRV) and adenovirus. Its efficiency can be affected by the integrity of viral genome and the linearization sites.

**Methods:**

In the study, we described a simple approach to isolate the viral DNA with high genomic integrity for large DNA viruses and a time-saving method to generate recombinant PRVs. Several cleavage sites in the PRV genome were investigated by using the EGFP as a reporter gene for identification of PRV recombination.

**Results:**

Our study showed that cleavage sites of XbaI and AvrII are ideal for PRV recombination which showed higher recombinant efficiency than others. The recombinant PRV-EGFP virus can be easily plaque purified in 1–2 weeks after the transfection. By using PRV-EGFP virus as the template and XbaI as the linearizing enzyme, we successfully constructed the PRV-PCV2d_ORF2 recombiant virus within a short period by simply transfecting the linearized PRV-EGFP genome and PCV2d_ORF2 donor vector into BHK-21 cells. This easy and efficient method for producing recombinant PRV might be adapted in other DNA viruses for the generation of recombinant viruses.

## Introduction

Pseudorabies virus (PRV) is a causative agent of Aujeszky's disease or pseudorabies that can cause reproductive failure characterized by abortion, embryonic death, mummification, and stillbirths. It also causes central nervous system problems, respiratory distress, and weight loss in pigs (Card et al., 1995; Guerin and Pozzi, 2005; Yin et al., 2012; Deng et al., 2022; Zheng et al., 2022). The virus belongs to the Herpesviridae family and has a double-stranded linear DNA genome. The genome of PRV is approximately 141–145 kb long which encodes at least 70 different proteins. A total of 11 different envelope glycoproteins of PRV have been identified, namely, gB, gC, gD, gE, gG, gH, gI, gK, gL, gM, and gN (Dietz et al., 2000; Klupp et al., 2004). The glycoproteins gB gD, gH, gL, and gK were identified as the essential proteins of PRV that are necessary for virus attachment to the host cell surface. The other glycoproteins such as gC, gE, gG, gI, gM, and gN are considered non-essential for viral entry and replication in which foreign genes can be inserted stably (Schmidt et al., 2001; Vallbracht et al., 2018). It has been reported how attenuated PRV can be a useful vector to develop recombinant vaccines for protection against both pseudorabies and other diseases (Thomsen et al., 1987; Freuling et al., 2017; Feng et al., 2020; Tong et al., 2020; Zheng et al., 2020).

PRV Bartha-K61 is an attenuated PRV vaccine strain in which complete gE and partial gI genes have been deleted. The vaccine strain was developed in Hungary and produced by extensive passage *in vitro*. It can grow well in pig kidney cells (PK-15 cells), baby hamster kidney fibroblast cells (BHK-21 cells), chicken eggs, and chicken embryo fibroblast cells (CEF cells) (Dong et al., 2014). As a marker vaccine, the Bartha-K61 vaccine has played a significant role in the prevention of PRV and differential diagnosis of wild-type viruses from vaccine strain due to its safety and immunogenicity in pig vaccination (An et al., 2013; Wang et al., 2014; Delva et al., 2020). It is still widely used in many countries, including China. To date, there are several ways to generate recombinant PRV, co-transfection of plasmid DNA containing homologous arms and virus or viral genome directly (Tong et al., 2020; Zheng et al., 2020; Tan et al., 2022) and CRISPR/Cas9-mediated homologous recombination (Tang et al., 2016; Feng et al., 2020). Although conventional homologous recombination methods provide a convenient way to produce recombinant viruses and recombinant vaccines, the efficiency of recombination including the plaque purification of the recombinant virus requires several rounds of screening and will likely consume valuable time. In the present study, we described an easy and efficient method for the isolation of PRV genome DNA intact and the construction of recombinant PRV Bartha-K61 virus. To demonstrate the application of the established method, the capsid protein gene (ORF2) of PCV2d (a variant strain of porcine circovirus type 2, characterized by severe respiratory disease complex in pigs, which has become a predominant genotype circulating in swine herds in many countries) was amplified and inserted into the genome of PRV Bartha-K61.

## Materials and methods

### Cells and virus

PK-15 and BHK-21 cells were purchased from the American Type Culture Collection (ATCC, VA, United States) and cultured in Minimum Essential Medium (MEM; Gibco, MA, United States) or Dulbecco's modified Eagle's medium (DMEM; Gibco, MA, United States), supplemented with 10% fetal bovine serum (FBS; Atlanta Biologicals, GA, United States) and 1x antibiotic-antimycotic (Gibco, MA, United States) at 37 °C within a 5% CO_2_ incubator. PRV Bartha-K61 strain was kindly provided by Professor Enquist (Princeton University). It was propagated in PK-15 cells and kept in liquid nitrogen until use.

### Extraction of viral DNA

To obtain a complete viral genome, PK-15 cells were plated in a T75-mm flask at a concentration of 5 × 10^5^ cells/flask and grown overnight to a confluence of 80–90%. The growth medium was replaced with 12 ml of fresh maintenance medium (MEM containing 2% FBS), and the cells were infected with PRV at a multiplicity of infection (MOI) of 0.5 PFU/cell. At 24 h post-infection, the culture medium was removed, and the cells were washed three times with 10 mL phosphate buffered saline (PBS). An additional 5 mL of PBS was added to the flask, and the cells were scraped into a 15-ml tube. After centrifugation at 2,000 x *g* for 20 min at 4°C, the cell pellet was resuspended in 1 ml lysis buffer solution (0.5% SDS, 10 mmol/L Tris-HCl pH 7.8, 5 mmol/L EDTA, 10 μg/ml RNase, and 50 μg/ml proteinase K) and incubated at 37°C in a water bath for 2–3 h. After centrifugation at 2,000 x *g* for 20 min at 4°C, the supernatant was collected in a new tube. The viral DNA in the supernatant was extracted with equal volumes of the UltraPure™ phenol:chloroform:isoamyl alcohol solution (25:24:1, v/v/v, Thermo Fisher Scientific, MA, United States) three times. The clear upper phase was transferred to a new 5-mL tube. In total, 2 volumes of ice-cold 100% ethanol and 1/10 volume of 3M sodium acetate (NaAc) at pH 5.2 were added to the tube, which was mixed by inverting the tube gently 8–10 times. The tube was then placed on ice for 10 min to separate the genomic DNA. A white floccule was obviously observed in the tube, which was the viral DNA. We carefully took the DNA using a sterile pipette tip or disposable inoculation loop and blotted the excess liquid, allowing it to dry for 5–10 min at room temperature. The viral DNA was resuspended in 200–500 μl TE buffer and maintained at 4°C for later use.

### Construction of plasmids

**Figure 1:**
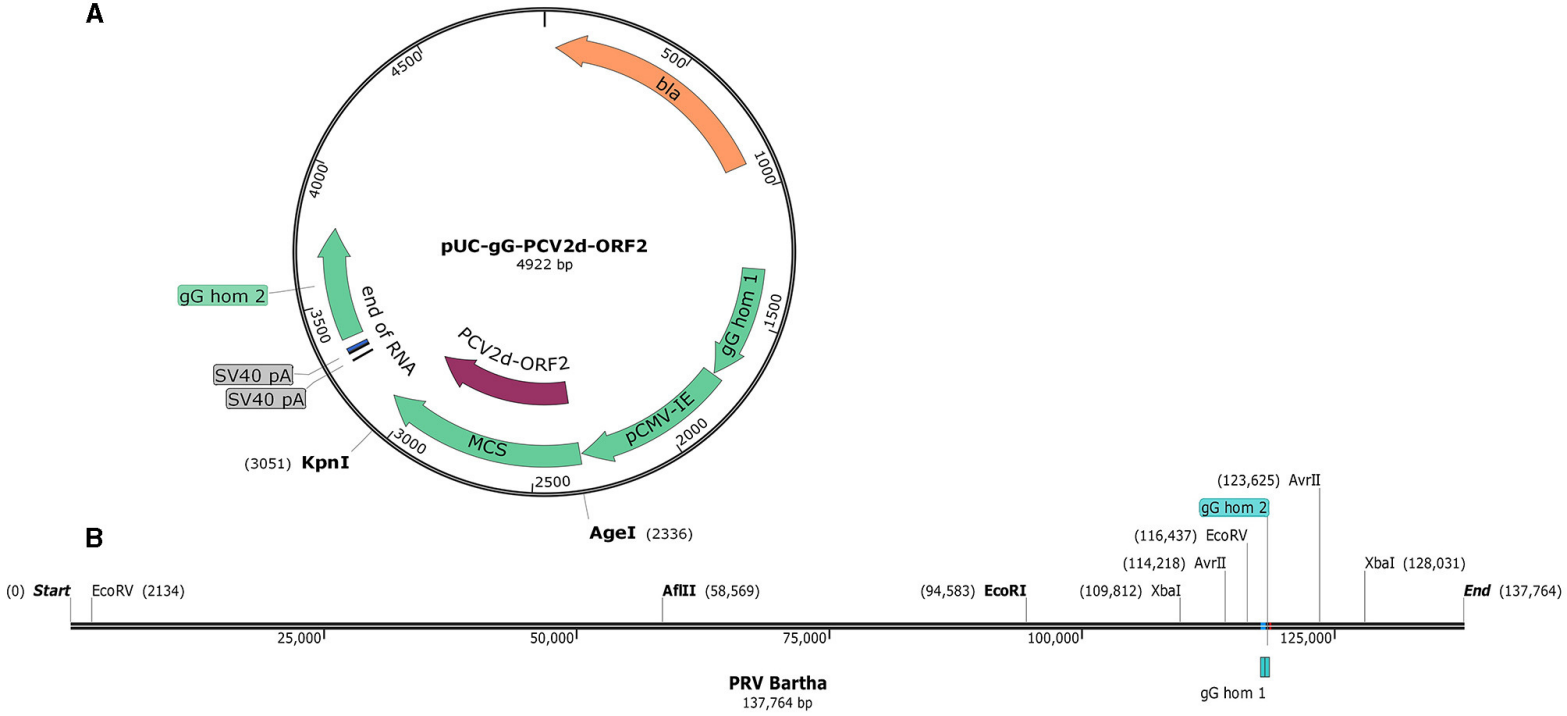
The map of the pUC-gG-MCS vector and the genome of PRV Bartha. **(A)** gG hom1 and gG hom2 are the two recombinant arms in the gG locus of PRV Becker. **(B)** Restriction enzyme analysis of the PRV Bartha genome, unique and dual cutters were listed and used in this study.

The pUC-gG-MCS (pUG) vector was constructed by Jens B. Bosse (Professor Enquist Lab, Princeton University). It was derived from pUC57 plasmid by inserting 850 bp of homology into the surroundings of the PstI site in the gG gene locus of the PRV Becker strain. For convenient insertion of exogenous genes, a pCMV-IE-MCS-SV40pA cassette was inserted between the two recombinant arms (Figure 1A). To verify the recombinant plasmid system and facilitate plaque visualization, the EGFP gene was cloned into pUG between the restriction sites of AgeI and KpnI to generate the plasmid pUG-EGFP. To further confirm the system and generate the recombinant virus, another plasmid pUG-PCV2d_ORF2 holding the PCV2d ORF2 gene was constructed. The PCV2d ORF2 gene was inserted into the same sites as the EGFP gene.

### Generation of recombinant virus

To investigate the efficiency of generation recombinant viruses and the chances of productive integration, different restriction enzymes were used to linearize the viral DNA according to the analysis of the viral genome (Figure 1B). Six groups with different transfection strategies were compared separately (Table 1). All linearized viral DNA and plasmids were precipitated with ethanol/NaAc as per the above description before the transfection step.

**Table 1:** Different groups of the transfection.

**Groups**	**Linearization of viral DNA**	**Linearization of plasmid**	**Transfection complex mixture**
A	EcoRI	HindIII	Linearized viral DNA + linearized plasmid
B	EcoRV	HindIII	Linearized viral DNA + linearized plasmid
C	XbaI	HindIII	Linearized viral DNA + linearized plasmid
D	AvrII	HindIII	Linearized viral DNA + linearized plasmid
E	XbaI	___	Linearized viral DNA + plasmid
F	___	HindIII	Virus + linearized plasmid

Viral genomes and plasmids were treated or non-treated with different restriction enzymes.

For transfection, BHK-21 cells were seeded into 6-well plates at 5 × 10^5^ cells/well so that the monolayers could be 80–90% confluent on the following day. In total, 3 μg of digested plasmid pUG-EGFP was co-transfected with 1.5 μg of linearized PRV genomic DNA using Lipofectamine 3000 (Thermo Fisher Scientific, MA, United States), according to the manufacturer's instructions. Fluorescent EGFP and CPE of the cells were checked daily under a fluorescent microscope with an objective lens of 20× magnification.

### Plaque purification

After 1 or 2 days of incubation at 37°C, the single plaques were marked on the underside of the 6-well plate using a fine-tip marker pen under a fluorescence microscope. For the generation of the recombinant PRV, either viral plaques with fluorescence were selected (PRV-EGFP) or viral plaques without fluorescence signals were selected (PRV-PCV2d_ORF2). All marked plaques were picked separately from a 1.5-ml tube containing 200 μl DMEM using a sterile Pasteur pipette, and then the viral plaques were labeled and stored at −80 °C as stocks for the next passage. After 2 to 3 rounds of plaque purification, the selected plaques were passaged on PK-15 cells, and the cultured recombinant viruses were subjected to further analysis.

### RT-PCR

Total cellular RNA of different plaque isolates was extracted using the commercially available viral nucleic acid extraction kit (IBI Scientific, IA, United States). The first-strand cDNA was prepared using a ProtoScript^®^ first strand cDNA synthesis kit (New England Biolabs, MA, United States), according to the manufacturer's instructions. To confirm the recombinant virus PRV-PCV2d_ORF2, the inserted fragment of ORF2 was verified using PCR with the PCV2d ORF2 special primers (Forward primer: 5′-ACCGGTGCCACCATGACGTATCCAAGGAGGCG-3′, reverse primers 5′-GGTACCTCACTTAGGGTTAAGTGGGG-3′).

### Immunofluorescence assay

PK-15 cells were dispensed into a 96-well plate and infected with PRV-PCV2d_ORF2 at an MOI of 1 in a final volume of 200 μl for 24 h. The cells were washed three times with PBS and fixed in cold methanol for 20 min at −20 °C. After fixation, the cells were permeated with 0.1% Triton X-100 at room temperature for 15 min and incubated with 5% FBS for 1 h at 37 °C. The cells were then incubated with anti-PCV2 capsid MAb (RTI, PA, United States) for 2 h at 4°C. After three washes with PBS, the cells were subjected to immunofluorescence staining with Alexa Fluor 488 goat anti-mouse IgG secondary antibody (Thermo Fisher Scientific, MA, United States) for 1 h at room temperature. Following three washes with PBS, the fluorescence signal was detected under a fluorescent microscope.

### Western blot

PK-15 cells were inoculated with PRV-PCV2d_ORF2 for 24 h in a 6-well plate. Cell lysates were separated using SDS-polyacrylamide gel electrophoresis (SDS-PAGE) with a gradient concentration of acrylamide (12%) followed by transfer onto nitrocellulose membranes. The membrane was blocked with 5% non-fat milk in PBS for 1 h and incubated with a mouse anti-PCV2 capsid MAb (RTI, PA, United States) overnight at 4°C. The following day, the membrane was incubated with a solution of horseradish peroxidase-conjugated rabbit anti-mouse IgG (Thermo Fisher Scientific, MA, United States) in PBS containing 1% non-fat milk for 1 h at room temperature. After incubation with SuperSignal West Pico chemiluminescent substrate (Thermo Fisher Scientific, MA, United States) for 5 min, the blots were analyzed with an imaging system.

## Results

### Generation of recombinant virus PRV-EGFP

**Figure 2:**
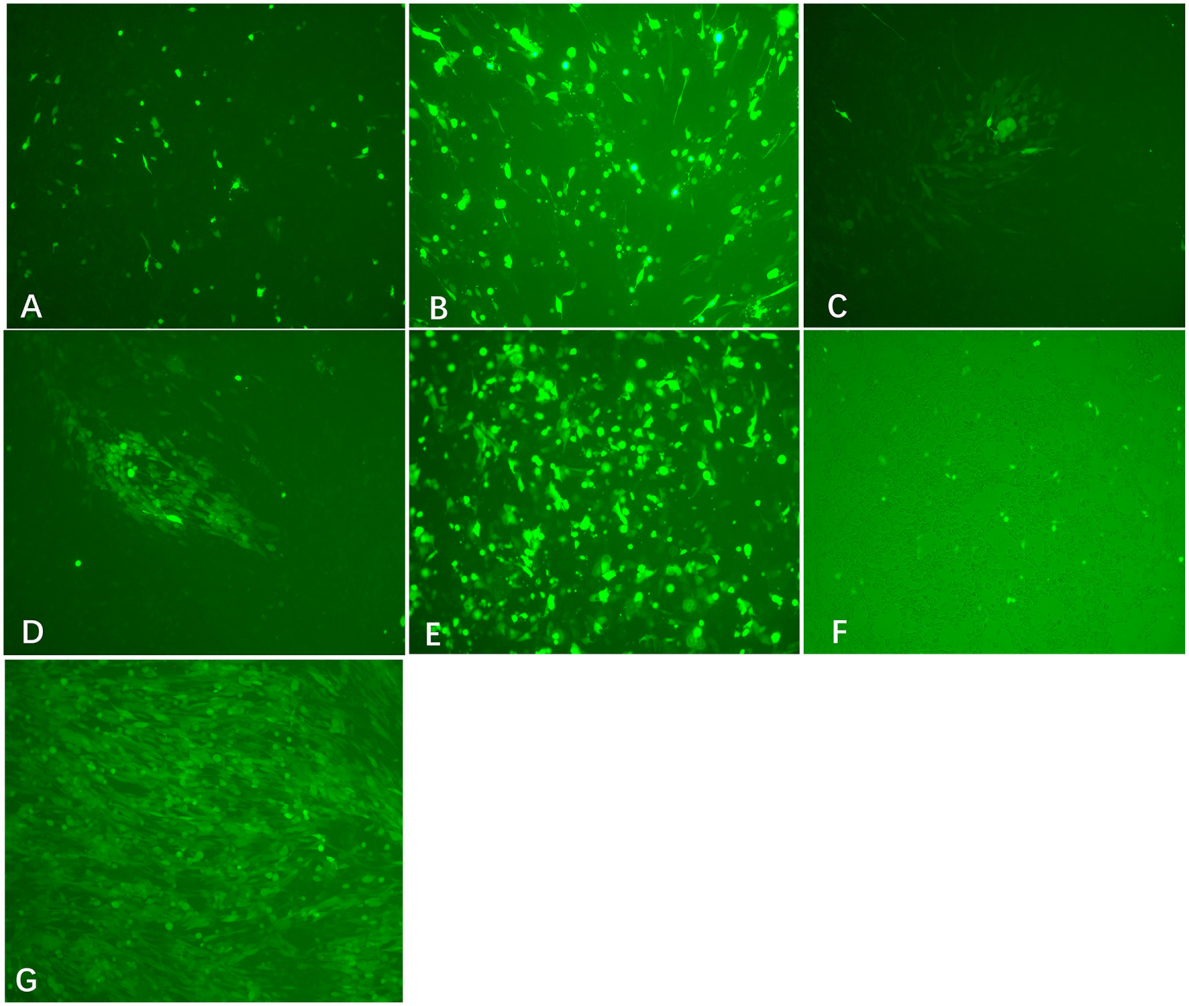
Transfection results of different treated viral DNA and plasmids (200×). Co-transfection of EcoRI-treated viral DNA + HindIII-treated pUG-EGFP **(A)**, EcoRV-treated viral DNA + HindIII-treated pUG-EGFP **(B)**, XbaI-treated viral DNA+ HindIII-treated pUG-EGFP **(C)**, AvrII-treated viral DNA + HindIII-treated pUG-EGFP **(D)**, XbaI-treated viral DNA + pUG-EGFP plasmid **(E)**, and virus + HindIII-treated pUG-EGFP **(F)** into BHK-21 cells, respectively. At 24 h after transfection, the expression of EGFP was observed in each group, but virus plaques were detected only in groups C and D. **(G)** The recombinant virus PRV-EGFP was obtained by plaque purification (200×).

Viral DNA was extracted from PRV Bartha-K61 strain-infected PK-15 cells. To facilitate plaque visualization, we cloned the EGFP gene into the pUC-gG-MCS (pUG) vector between the restriction sites of AgeI and KpnI to generate the plasmid pUG-EGFP. Co-transfection of the XbaI/AvrII linearized viral DNA and HindIII linearized pUG-EGFP into BHK-21 cells can produce obvious CPE and fluorescence signal at 24 h post-transfection (Figure 2G). The plaque purification of the recombinant viruses can be performed directly after the transfection. After two or three rounds of plaque picking, we successfully obtained the recombinant virus PRV-EGFP.

### Selection of cleavage sites significantly affects the efficiency of recombination

To investigate the impact of cleavage sites on the efficiency of recombination, we linearized the viral DNA by different restriction enzymes. Co-transfection of linearized viral DNA with non-linearized plasmid pUG-EGFP caused an observable EGFP signal after transfection (Figure 2E). However, most of the fluorescence disappeared after the second round passage. When co-transfecting linearized viral DNA with linearized plasmid pUG-EGFP, expression of EGFP in cells can be observed in the EcoRI or EcoRV-treated viral DNA group. However, CPE or viral plaques were not easily detected after transfection (Figures 2A, B). Most interestingly, only the viral genome that was digested by XbaI or AvrII can cause obvious CPE and plaques after co-transfection with the linearized plasmid pUG-EGFP (Figures 2C, D). The recombinant efficiency of the AvrII-treated viral genome is higher than that of the XbaI-treated viral genome, which can produce more viral plaques. This indicates that the closer the linearized incision is to the ends of the recombination arm, the higher the recombination efficiency that will be generated.

### Generation of the recombinant virus PRV-PCV2d_ORF2

**Figure 3:**
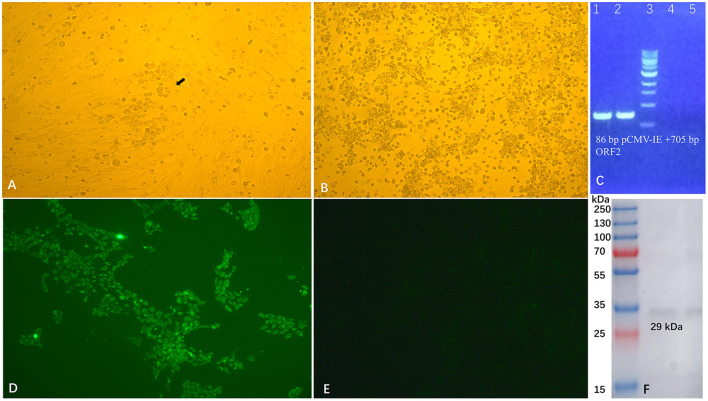
Generation and identification of recombinant PRV-PCV2d_ORF2. The recombinant PRV-ORF2 was purified by plaque picking from BHK-21 cells (200 ×) **(A)**, and the purified virus was then propagated in PK-15 cells **(B)**. RT-PCR **(C)**, IFA **(D)**, and Western blot **(F)** were used to confirm the expression of PCV2d capsid protein. Cells infected with PRV-PCV2d_ORF2 developed immunofluorescence and the expression of capsid protein could be detected by PCV monoclonal antibody, and cells infected with Bartha did not show immunofluorescence **(E)**.

The strategy to efficiently construct recombinant virus PRV-PCV2d_ORF2 is using the genome of the PRV-EGFP virus as the template and replacing the EGFP gene with PCV2d_ORF2 using the homologous recombination approach. We inserted the PCV2d ORF2 gene into the vector pUG to generate plasmid pUG-PCV2d_ORF2. As expected, plaques formed 24 h post-transfection by co-transfecting of XbaI-treated (compared with AvrII, XbaI is an economical site) genome DNA of PRV-EGFP virus and HindIII-treated plasmid pUG-PCV2d_ORF2 into BHK-21 cells. After two rounds of viral plaque purification (Figure 3A), the purified viruses without bring fluorescence were passaged on PK-15 cells (Figure 3B). RT-PCR (Figure 3C), sequencing, IFA, and Western blot (Figures 3D–F) results showed that we successfully obtained the recombinant virus PRV-PCV2d_ORF2.

## Discussion

Homologous recombination is a type of genetic recombination in which the genetic material of the virus, eukaryote, or bacteria is exchanged naturally between two molecules of DNA that contain similar recombinant arms. Over the past few decades, it has been used extensively in the construction of recombinant adeno-associated virus (Fisher et al., 1997; Jacob et al., 2020), poxvirus (Fisher et al., 1997; Wyatt et al., 2015), and herpesvirus (Wilkinson and Weller, 2003; Boscheinen et al., 2019). It is a powerful tool to precisely manipulate the genome for producing a new gene or virus according to the experimental need. There were a variety of ways to produce recombinant PRV according to the previous reports (Takashima et al., 2002; Lin et al., 2005; Lerma et al., 2016; Tang et al., 2016). However, it is very time consuming to generate recombinant PRVs by using the limited dilution method. The strategy mentioned in this report, i.e., makes the plaque purification possible by monitoring EGFP which can be replaced in the future, significantly shortening the time for constructing recombinant PRVs.

Previous studies have reported that linearizing viral DNA at the desired insertion site before transfection can enforce homology-directed repair (HDR) by recombination with the co-transfected plasmids. To achieve this, a transfer virus expressing EGFP must be generated first by co-transfecting plasmid with PRV or PRV genome. Two unique restriction sites were designed and flanked on both sides of the EGFP-coding sequence, then the unique restriction sites could be used between the plasmid expressing a gene of interest and the PRV-EGFP genome (Klingbeil et al., 2014). However, the step for preparing recombinant virus PRV-EGFP may require several rounds and weeks of plaque purification (Zhao et al., 2020). In recent years, the CRISPR/Cas9 system has also been widely used in homology-directed repair (HDR), this approach can be used to introduce desired sequences by homologous recombination (Hirohata et al., 2019). Undeniably, the CRISPR/Cas9 technology has emerged as a powerful tool that enables ready modification of the mammalian genome and accelerates biological and medical research *in vivo*. However, the efficiencies of CRISPR/Cas9-mediated homologous recombination are still limited by the sizes of targeted chromosomal regions and donor DNAs. DNA repair may cause deletion, insertion and mutation in CRISPR/Cas9 target sites for homologous recombination, and to avoid this, sgRNA should be designed at uncritical regions, such as introns (Zhang et al., 2020). In addition, promiscuous cleavage of off-target sites remains a major concern in the application of the CRISPR/Cas9 technology (Lin et al., 2016; Rose et al., 2020). In this study, the viral DNA was digested with restriction enzymes cleaving at one (EcoRI) or more sites (AvrII, EcoRV, and XbaI) in the genome. After transfection, we can restore the infectious full-length genome, which is quite efficient.

Previous studies have found that the topology of DNA can affect transfection efficiency. Although linearized DNA may have a lower efficiency of transfection compared with the circular DNA, it can improve the efficiency of generating stable transfected cells and enhance the recovery of recombinant viruses (Kitts et al., 1990; von Groll et al., 2006; Hsu and Uludag, 2008; Stuchbury and Munch, 2010). The efficiency of the recombination was up to 10-fold higher than that of co-transfections with circular DNA when using linearized plasmids to produce recombinant baculovirus (Kitts et al., 1990). However, the site of cleavage also played an important role in both transient and stable transfection efficiency (Stuchbury and Munch, 2010). In the present study, we compared the effects of cleavage sites on recombination efficiency. The sites of XbaI and AvrII were close to the recombinant arms and had high recombination efficiency when using these sites to cut the viral genome, which suggests that the closer to the recombinant arm, the higher the obtained efficiency will be. The different outcomes of transfection experiments with EcoRI, EcoRV, XbaI, and AvrII-digested PRV DNA might be due to the different relevance of the affected genome positions and their sensitivity to erroneous NHEJ (non-homologous end joining) repair. XbaI and AvrII cut sites within the inverted repeat regions (IR-S and TR-S) of the genome and possibly correct repair of one copy might be sufficient to restore infectivity.

Furthermore, the integrity of the viral genome is crucial for producing recombinant viruses. We have tried multiple methods to isolate the whole viral genome including commercially available viral nucleic acid extraction kits (IBI Scientific, IA, United States) and different ways to precipitate the virus particles including the PEG precipitation. None of them was able to obtain an intact viral DNA genome. The method described here was the most convenient and did not require a special reagent or instrument. We also provide insight that this method can be used for adenovirus, poxvirus, and other herpesviruses for large viral DNA genome isolation.

## Data availability statement

The original contributions presented in the study are included in the article/supplementary material, further inquiries can be directed to the corresponding authors.

## Author contributions

LW and JS designed and supervised the project. JR, LW, and JS collected literature, drafted the original manuscript, and analyzed the data. JR, RM, and CC performed the experiment. All authors contributed to the article and approved the submitted version.
